# Changes in Serum Concentrations of Bone Turnover Markers in Healthy Pregnant Women

**DOI:** 10.1155/2023/8466349

**Published:** 2023-12-16

**Authors:** Yiduo Zhang, Ruiying Li, Jing Zhang, Wenjie Zhou, Fan Yu

**Affiliations:** ^1^Department of Laboratory Medicine, West China Second University Hospital, Sichuan University, Chengdu, Sichuan, China; ^2^Key Laboratory of Birth Defects and Related Diseases of Women and Children (Sichuan University), Ministry of Education, Chengdu, Sichuan, China

## Abstract

**Background:**

Changes in bone metabolism during pregnancy have not received sufficient attention because of the lack of effective screening tools. Bone turnover markers (BTMs) could reflect the changes of bone metabolism. Currently, reference intervals for bone metabolism during normal pregnancy are inconclusive. This study aimed to determine reference intervals for BTMs in pregnant women taking prenatal care and to facilitate clinical research on diseases affecting bone metabolism during pregnancy.

**Methods:**

We surveyed 120 low-risk pregnant women attending routine antenatal care from January 2020 to March 2020. The serum levels of procollagen type I N-propeptide (PINP), N-terminal osteocalcin (N-MID), and C-terminal telopeptide of type I collagen (*β*-CTX) were measured in the first trimester (<13 weeks), second trimester (14–27 weeks), and third trimester (>28 weeks). Reference intervals for BTMs during pregnancy were analyzed. The Kruskal–Wallis test and paired *t*-test are used to analyze differences between groups. Spearman correlation coefficients expressed the measure of linear association.

**Results:**

The bone resorption marker *β*-CTX in third trimester increases compared to the first trimester and the second trimester (*P* < 0.001, *P* < 0.001). The bone formation markers PINP and N-MID were decreased from the first trimester to the second trimester (*P* = 0.01, *P* < 0.001) and then raised from the second trimester to the third trimester (*P* < 0.001, *P* < 0.001). Two indices of bone turnover rate, *β*-CTX/PINP and *β*-CTX/N-MID, were increased from the first trimester to the second trimester (*P* < 0.001, *P* < 0.001) and then decreased from the second trimester to the third trimester (*P* = 0.02, *P* < 0.001).

**Conclusion:**

This study established reference intervals for BTMs in pregnant women and observed the changes in BTMs during the different trimesters of pregnancy. The present findings can help in clinical monitoring of the effects of pregnancy diseases on the bone metabolism of pregnant women.

## 1. Introduction

Osteoporosis is a disease characterized by low bone mass and microarchitectural deterioration of bone tissue and is often asymptomatic before the development of bone fractures. Pregnancy and lactation-associated osteoporosis is a rare and severe form of osteoporosis that results in severe low back pain during late pregnancy or lactation and causes spontaneous fractures, most commonly multiple vertebral fractures [1]. Although bone mineral density (BMD) assessment is frequently employed for osteoporosis diagnosis based on dual-energy X-ray absorptiometry measurements, the teratogenic nature of X-rays means that such BMD assessment is prohibited for osteoporosis detection in pregnant women. Consequently, developing a safe and effective method for evaluating bone health status in pregnant women is necessary. Bone turnover markers (BTMs) are released explicitly during bone resorption and bone formation and provide dynamic data on bone growth. There is evidence that BTMs, including the serum levels of procollagen type I N-propeptide (PINP), N-terminal osteocalcin (N-MID), and C-terminal telopeptide of type I collagen (*β*-CTX), can reflect the balance between bone formation and bone resorption and have important reference value for clinical diagnosis and dynamic treatment evaluation in bone diseases [2–4].

A recent study highlighted BTMs' reference intervals for healthy premenopausal females and teenagers [5–8]. However, due to the influence of the fetus on the bone metabolism of pregnant women, the bone metabolism of pregnant women is more complicated, and these reference intervals do not apply to pregnant women. The main objectives of this cross-sectional study were to define reference intervals for BTMs in pregnant women and to investigate the variety of BTMs in different stages of pregnancy that can be performed. The study could provide scientific BTM data for comparison between healthy pregnant women and pregnant women with the disease which affects bone metabolism.

## 2. Methods

### 2.1. Participants

We selected 186 low-risk pregnant women attending routine antenatal care from January 2020 to March 2020. All subjects should follow medical advice and take calcium tablets including 230 mg calcium and 10 *μ*g vitamin D3 (Caltrate, Pfizer, USA) and multivitamin tablets including 125 mg calcium and 5 *μ*g vitamin D3 (Elevit, Bayer, Germany). Calcium tablets have been replenished daily during pregnancy, taking one daily pill. Multivitamin tablets are prescribed for the first three months of pregnancy, taken once per day, and will not be prescribed in the future. None of the subjects had any limitations in the diet. One hundred twenty healthy women who underwent examination at the physical examination center were selected as the control group. All subjects are Han population.

**Figure 1:**
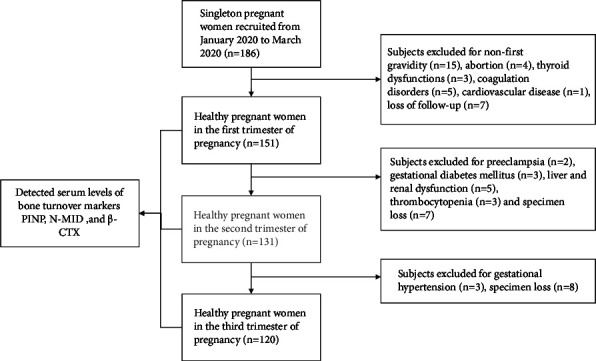
Flowchart of the study showing the steps to select the reference population. PINP, procollagen type I N-propeptide; N-MID, N-terminal osteocalcin; *β*-CTX, C-terminal telopeptide of type I collagen.

The exclusion criteria were previous pregnancy history; lifestyle factors such as smoking and alcohol consumption; chronic diseases such as liver dysfunction, diabetes, hypertension, thyroid diseases, and autoimmune diseases; pregnancy complications; and mental disorders. As shown in Figure 1, 120 women were randomly selected as the study subjects among the women who met the inclusion criteria. General background characteristics for the 120 pregnant women were collected, including age, height, weight, and gestational weeks. Body mass index (BMI) was calculated as follows: weight (kg)/height squared (m^2^).

The participants were not involved in the recruitment and conduct of the study. We obtain diagnostic information from the laboratory information system. The participants have been informed and accepted that the remaining serum samples can be used for scientific research experiments. The study was reviewed and approved by the Ethics Committee of our hospital, and all participants signed the informed consent form.

### 2.2. Sampling Procedures

Gestational age was calculated from the first day of the last normal menstrual period and classified at the time of blood sampling as first trimester (≤13 weeks of pregnancy), second trimester (14–27 weeks of pregnancy), or third trimester (≥28 weeks of pregnancy). The pregnant women were instructed to fast overnight for a minimum of 8 h, and fasting blood samples were collected in vacuum tubes with a gel separator plus clot activator between 08:00 and 10:00. The whole-blood samples were centrifuged immediately (3500 rpm for 10 min), and the serum samples were stored at −80°C until analysis.

### 2.3. Routine Biochemistry Indicators and BTM Measurements

Serum 25(OH)D concentration was determined using LIAISON (DiaSorin, Saluggia, Italy). This direct competitive chemiluminescence immunoassay recognizes 25 (OH) vitamin D2 and 25 (OH) vitamin D3 and is fully automated using a LIAISON platform. Serum calcium (Ca), phosphorus (Pi), and alkaline phosphatase (ALP) were detected by ADVIA 2400 (Siemens, Berlin, Germany). Serum PINP, N-MID, and *β*-CTX were measured by a fully automated electrochemiluminescence system (E411; Roche Diagnostics, Basel, Switzerland) using a single-step sandwich electrochemiluminescence immunoassay. Standard reagents, calibrators, and quality control material manufactured were used during the sample testing process. All analytical measurements were performed according to the manufacturer's instructions regarding preventive maintenance, function checks, calibration, and quality control of tests and equipment.

### 2.4. Statistical Analysis

**Figure 2:**
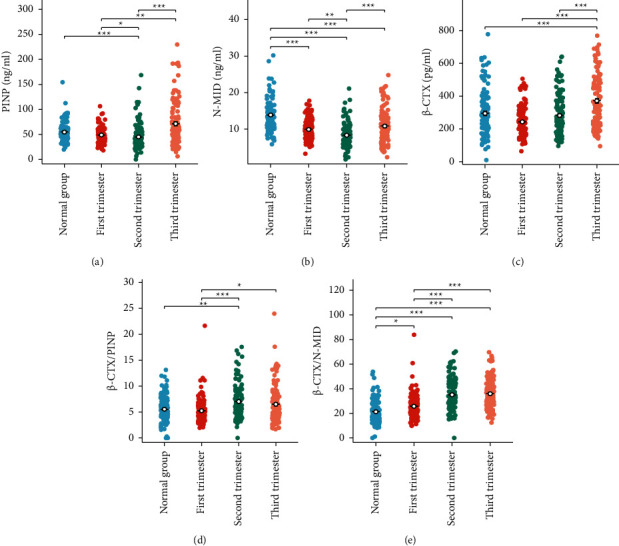
The trend of bone turnover markers in different stages of pregnancy: PINP (a), N-MID (b), *β*-CTX (c), *β*-CTX/PINP ratio (d), and *β*-CTX/N-MID ratio (e) at each trimester during pregnancy. Statistics were calculated by the Kruskal–Wallis test. ns, *P* ≥ 0.05; ^*∗*^*P* < 0.05; ^*∗∗*^*P* < 0.01; ^*∗∗∗*^*P* < 0.001. PINP, procollagen type I N-propeptide; N-MID, N-terminal osteocalcin; *β*-CTX, C-terminal telopeptide of type I collagen.

SPSS 25.0 software (IBM, Armonk, NY, USA) was used for all statistical analyses, and the Kolmogorov–Smirnov Z method was employed to determine the normality of the data distributions. Following the CLSI C28-A3 guidelines, the PINP, N-MID, and *β*-CTX reference intervals were represented using a nonparametric percentile method. In brief, each reference interval had upper and lower limits of 2.5th and 97.5th percentiles, respectively. The Kruskal–Wallis test was used to analyze the variation in BTMs and biochemical index for different gestational ages, and the paired *t*-test was used to analyze the variation in general background characteristics of the study subjects. Spearman correlation coefficients expressed the measure of linear association. Values of *P* < 0.05 were considered to indicate statistical significance. For visualization, Figure 2 is drawn with ggplot2 of R language (https://www.r-project.org/).

## 3. Results

### 3.1. The General Characteristics and Biochemical Indices of Study Participants

As shown in Table 1, the pregnant women's BMI, 25(OH)D, and ALP gradually increased with increasing gestational age. There were significant differences in 25(OH)D and ALP between the first trimester and the second trimester (*P* < 0.001) and between the first trimester and the third trimester (*P* < 0.001). 25(OH)D and ALP in the third trimester were significantly higher than those in the second trimester (*P* < 0.001). Serum calcium in the first trimester was more elevated than the second trimester (*P* < 0.001) and the third trimester (*P* < 0.001). The concentration of Pi in the first trimester was lower than that of the third trimester (*P* = 0.01).

### 3.2. Differences in BTM Levels at Different Stages of Pregnancy

As shown in Table 2, all markers of bone formation studied exhibited a similar pattern of change with time. Serum PINP and N-MID decreased significantly in the second trimester and increased significantly in the third. Serum PINP was substantially lower in the second trimester compared with the first trimester (*P* = 0.01) and the third trimester (*P* < 0.001), and there was also a significant difference between the first trimester and the third trimester (*P* = 0.007) (Figure 2(a)).

As illustrated in Figure 2(b), serum N-MID was significantly decreased in the second trimester compared with the first trimester (*P* < 0.001) and the third trimester (*P* < 0.001). However, there was no significant difference between the first and second trimesters (*P* = 0.109).

The bone resorption marker *β*-CTX was significantly increased in the third trimester compared with the first trimester (*P* < 0.001) and the second trimester (*P* < 0.001), but there was no significant difference between the first trimester and the second trimester (*P* = 0.105) (Figure 2(c)).

Two indices of bone turnover rate, *β*-CTX/PINP and *β*-CTX/N-MID, were significantly lower in the first trimester compared with the second trimester (*P* < 0.001, *P* < 0.001) and the third trimester (*P* = 0.02, *P* < 0.001) (Figures 2(d) and 2(e)).

### 3.3. Reference Intervals for BTMs at Different Stages of Pregnancy

Lower and upper limits of reference intervals for BTMs were derived from the 2.5th and 97.5th percentiles, respectively, as shown in Table 3.

## 4. Discussion

Bone metabolism couples bone formation and bone resorption in different parts of the body. Bone formation by osteoblasts involves organic and inorganic matrix deposition, promoting bone matrix formation and increasing bone salt deposition [9]. In contrast, bone resorption by osteoclasts involves bone matrix removal and bone salt dissolution. BTMs are byproducts of bone resorption and formation and represent specific molecules and molecular fragments formed during bone remodeling.

The mother's nutritional status and the nutrition the fetus requires vary. Inorganic salts, including calcium, phosphorus, and magnesium, were significantly higher in fetal plasma than in maternal plasma. During the first trimester of pregnancy, the cartilage skeleton forms at an early stage of embryonic development [10]. In the second and third trimesters, the development of fetal bone mainly involves mineralization, and the degree of fetal bone mineralization gradually increases [11, 12]. The level of bone turnover in pregnant women is different from that in non-pregnant women.

On the one hand, the maternal calcium responds to the needs of the fetus, while on the other hand, the increases in blood volume and glomerular filtration rate in the third trimester of pregnancy lead to an increase in calcium loss and a decrease in blood calcium concentration [13]. These factors promote passive and active bone formation during the third trimester of pregnancy, and the synthesis of osteoblasts increases. Timely and rapid monitoring of changes in bone metabolism indicators during pregnancy is helpful for clinical intervention through treatment measures [14].

PINP and procollagen-1 C-propeptide (P1CP) are bone formation markers synthesized as procollagen precursors [15, 16]. PINP has a longer circulating half-life period in vivo than P1CP and is a specific marker for bone formation and osteoblast activity. The serum concentration of PINP is proportional to the rate of bone formation. The International Osteoporosis Foundation and the International Federation of Clinical Chemistry and Laboratory Medicine recommend that serum PINP be used as a reference marker for bone formation [15]. Our findings are consistent with a study showing that serum PINP increases much later than serum bone resorption markers such as *β*-CTX, suggesting that the former is compensatory [17, 18]. Therefore, the Chinese National Health and Family Planning Commission recommends that pregnant women have a daily calcium intake of 1000 mg during the second trimester of pregnancy and increase their intake to 1200 mg from the third trimester of pregnancy to the end of lactation [19].

N-MID is a stable form of the osteocalcin carboxyl fragment secreted by mature osteoblasts and precisely reflects the status of bone formation [20]. Serum osteocalcin is regarded as a marker for the activity status of osteoblasts, primarily newly formed osteoblasts. The serum concentration of N-MID is consistent with that of PINP, and the serum level of N-MID in the second trimester of pregnancy is lower than that in the first and third trimesters. Sufficient blood calcium is required for fetal bone formation during early pregnancy [21]. Based on the characteristics of the traditional Chinese dietary structure, including low milk consumption and low dietary calcium intake, maternal calcium storage is often insufficient [22]. The activity of bone formation shows a downward trend from the first to the second trimester when calcium is mobilized from the maternal bone into the blood to contribute a significant component of the fetal requirement for calcium while also maintaining maternal calcium homeostasis. From the second trimester to the third trimester, the demand for calcium for fetal bone mineralization is at its maximum level [23]. At the same time, the production of N-MID requires vitamin D, and ultraviolet-B light is essential for synthesizing vitamin D. Reducing outdoor activities during late pregnancy may reduce skin exposure to ultraviolet-B light, affecting calcium absorption in the mother. This change further stimulates the compensatory increase in bone formation to maintain bone remodeling at a set level.

*β*-CTX is released into the blood after degradation of type I collagen during the bone resorption process by osteoclasts [24]. It is a sensitive marker that reflects osteoclast activity and is internationally recognized as a marker for bone resorption. Bone resorption increases with increasing stages of gestation and leads to bone loss in pregnant women. During the third trimester of pregnancy, maternal bone resorption peaks [25–27]. In the present study, we also found that healthy pregnant women exhibited significant increases in *β*-CTX in the first, second, and third trimesters as the gestational weeks progressed. During pregnancy, prolactin and estrogen increase calcium absorption in the intestine and kidney [9, 28]. The mother has no noticeable bone loss during the first trimester of pregnancy. However, in the second and third trimesters, the demand for bone mineralization in the fetus increases, and the rate of bone absorption increases in the mother, inevitably leading to bone loss.

Bone resorption and bone formation are tightly coupled and equivalent in healthy women. However, the markers of bone resorption and bone formation in populations can show individual differences, and the relationship between bone resorption and bone formation can be uncoupled. Therefore, the bone turnover rate is needed to evaluate bone metabolism. *β*-CTX/PINP ratio and *β*-CTX/N-MID ratio are associated with a negative bone remodeling balance, and they can be used to assess the effects of various diseases on bone metabolism and predict fracture risk [3, 29, 30]. The present study observed similar trends for the *β*-CTX/PINP ratio and *β*-CTX/N-MID ratio during pregnancy. Increased bone resorption suppresses bone formation from the first to the second trimester. Still, the compensatory increase in bone formation fails to match the initial increase in bone resorption, and thus bone resorption is dominant. From the second trimester to the third trimester, bone resorption is further aggravated, but bone formation compensates for the increase, and the ratios show a downward trend. These two indices also provide a reference for clinical judgment of bone metabolism in pregnant women.

Our results during pregnancy largely support previous studies reporting increasing plasma levels of 25(OH)D and ALP [26, 31, 32]. The significant increase in maternal plasma 25(OH)D is believed to be caused by daily drug supplements on the one hand and the increase of renal and extrarenal synthesis in the placenta on the other hand [33–35]. Serum tartrate-resistant acid phosphatase type 5b (TRACP 5b) is a bone resorption marker, and bone alkaline phosphatase (BAP) is a bone formation marker. During fetal growth from middle pregnancy to birth, TRACP 5b activity gradually decreased. In contrast, BAP activity steadily increased [36], which may lead to increased ALP in the mother.

ALP is a group of isoenzymes available in the placenta, intestinal, kidney, bone, and liver. In general, bone ALP contributes about half the total ALP. High activity of ALP correlates with more active bone metabolism [37]. During pregnancy, placenta ALP is released into the maternal circulation, and its plasma concentration increases as pregnancy proceeds, even exceeding the concentration of bone ALP [38]. In the correlation analysis between BTMs and biochemical indices, there was a positive correlation between *β*-CTX, PINP, and ALP, which proved that the more active the bone metabolism was, the higher the concentration of BTMs was (shown in Figure S1). ALP as a stand-alone to assess bone metabolism is far from accurate and needs to be comprehensively analyzed with bone formation and resorption indices.

This study has several limitations. Firstly, the sample evaluated is insufficient, so we are conducting a multicenter observational trial to get more data to strengthen statistical power. Secondly, we hypothesized that the effect of hormone levels on BTMs was consistent in all pregnant women. Next, we will analyze the possible correlation between circulating estrogen levels during pregnancy and the variation of BTMs. In addition, due to experimental methods, the detection results of some indicators, including bone ALP, are graded data, and we use total ALP to replace them. Although it will not affect the study's results, we will further refine our experimental results in the following research.

## 5. Conclusions

The present study aimed to clarify the changes in BTMs for pregnant women in Southwest China. Considering regional differences, the prevalence rate of osteoporosis in Southwest China is higher than that in other regions of China. Therefore, good management during pregnancy is necessary for both mother and fetus. Our study determined the changes in bone metabolism during pregnancy in Southwest China by detecting BTMs, to provide an essential reference for the clinical prevention of osteoporosis during pregnancy. The study findings could provide a basis for further clinical research on the effects of pregnancy complications on the bone metabolism of pregnant women.

## Figures and Tables

**Table 1:** The general characteristics and biochemical indices of subjects.

	Normal group (*n* = 120)	First trimester (*n* = 120)	Second trimester (*n* = 120)	Third trimester (*n* = 120)
Age (years)	27.5 ± 3.7	28.2 ± 2.6	28.3 ± 2.4	28.4 ± 2.4
BMI (kg/m^2^)	20.3 ± 2.8	22.0 ± 2.5	23.8 ± 2.3	25.7 ± 2.5
Gestational weeks	—	8.9 ± 1.5	20.1 ± 2.9	33.0 ± 3.1
25(OH)D (ng/ml)	15.7 (11.8, 23.1)	18.4 (11.3, 23.4)	23.3 (14.0, 31.8)^a^	31.6 (25.7, 39.5)^ab^
Ca (mmol/L)	2.40 (2.34, 2.45)	2.24 (2.20, 2.31)	2.19 (2.12, 2.27)^a^	2.19 (2.13, 2.24)^a^
Pi (mmol/l)	1.17 (1.07, 1.28)	1.18 (1.16, 1.31)	1.24 (1.13, 1.34)	1.29 (1.17, 1.37)^a^
ALP (U/l)	63 (52, 75)	46 (35, 60)	60 (52, 82)^a^	97 (85, 122)^ab^

^a^Compared to the first trimester, *P* < 0.05. ^b^Compared to the second trimester, *P* < 0.05. Data are presented as mean ± standard deviation or median (25–75%; interquartile range). Statistics were calculated by the paired *t*-test. BMI, body mass index; Ca, calcium; Pi, phosphorus; ALP, alkaline phosphatase.

**Table 2:** Analysis of the difference in BTM level at different stages of pregnancy.

	Normal group (*n* = 120)	First trimester (*n* = 120)	Second trimester (*n* = 120)	Third trimester (*n* = 120)
PINP (ng/ml)	53 (43, 62)	48 (40, 55)	39 (30, 51)^ab^	58 (39, 96)^bc^
N-MID (ng/ml)	13.4 (11.3, 15.7)	9.6 (7.7, 11.8)^a^	7.7 (6.1, 10.1)^ab^	10.6 (7.6, 13.0)^ac^
*β*-CTX (pg/ml)	264 (193, 376)	236 (172, 297)	268 (178, 353)	343 (231, 498)^abc^
*β*-CTX/PINP	5.3 (3.8, 7.0)	5.3 (3.6, 6.2)	7.1 (4.8, 8.9)^ab^	6.5 (4.1, 8.0)^a^
*β*-CTX/N-MID	19.6 (14.6, 25.4)	25.7 (17.8, 32.2)^a^	35.5 (28.4, 43.8)^ab^	35.9 (28.2, 41.9)^ab^

^a^Compared to the normal group, *P* < 0.05. ^b^Compared to the first trimester, *P* < 0.05. ^c^Compared to the second trimester, *P* < 0.05. Data are presented as median (25–75%; interquartile range). Statistics were calculated by the *Kruskal–Wallis* test. PINP, procollagen type I N-propeptide; N-MID, N-terminal osteocalcin; *β*-CTX, C-terminal telopeptide of type I collagen.

**Table 3:** Reference interval of BTMs at different stages of pregnancy.

		Lower	Upper	90% CI for lower limit	90% CI for upper limit
PINP (ng/ml)	First trimester	23	91	(18, 28)	(80, 106)
	Second trimester	12	115	(7, 21)	(90, 168)
	Third trimester	21	192	(6, 23)	(168, 229)

N-MID (ng/ml)	First trimester	5.3	16.5	(3.3, 5.7)	(15.1, 17.7)
	Second trimester	2.6	17.2	(1.7, 4.2)	(14.9, 21.0)
	Third trimester	3.9	21.5	(2.3, 5.0)	(20.0, 24.7)

*β*-CTX (pg/ml)	First trimester	112	462	(65, 123)	(445, 505)
	Second trimester	118	638	(97, 125)	(528, 972)
	Third trimester	149	698	(96, 163)	(656, 768)

*β*-CTX/PINP	First trimester	2.4	11.1	(2.0, 2.8)	(8.7, 21.4)
	Second trimester	2.8	16.2	(2.1, 3.4)	(14.2, 17.8)
	Third trimester	1.9	14.3	(1.7, 2.6)	(13.5, 23.8)

*β*-CTX/N-MID	First trimester	11.3	49.8	(9.9, 13.0)	(40.2, 83.2)
	Second trimester	15.8	62.3	(15.1, 18.4)	(59.6, 70.2)
	Third trimester	16.5	65.3	(12.5, 19.0)	(62.3, 69.5)

PINP, procollagen type I N-propeptide; N-MID, N-terminal osteocalcin; *β*-CTX, C-terminal telopeptide of type I collagen.

## Data Availability

The data used to support the findings of this study are included within the article.

## Abbreviations

BTMs: Bone turnover markers
PINP: Procollagen type I N-propeptide
N-MID: N-terminal osteocalcin
*β*-CTX: C-terminal telopeptide of type I collagen
BMD: Bone mineral density
BMI: Body mass index
TRACP 5b: Serum tartrate-resistant acid phosphatase type 5b
BAP: Bone alkaline phosphatase
P1CP: Procollagen-1 C-propeptide
ALP: Alkaline phosphatase
Ca: Calcium
Pi: Phosphorus.

## Supplementary Materials

Supplementary Materials Figure S1: scatterplots showing the relation between serum concentrations of BTMs and biochemical indices in healthy pregnant women.
