# Supplementary material for: Changes in Serum Concentrations of Bone Turnover Markers in Healthy Pregnant Women
Source: Int J Clin Pract. 2023 Dec 16;2023:8466349. doi: 10.1155/2023/8466349 (PMC10748724; doi:10.1155/2023/8466349)
Supplement: Supplementary Materials — Figure S1: scatterplots showing the relation between serum concentrations of BTMs and biochemical indices in healthy pregnant women. [file 8466349.f1.docx]

**Correlation between BTMs and biochemical indices**

As shown in Figure S1, β-CTX and PINP are positive correlation with 25(OH)D (*P*<0.001, *P*<0.001) and ALP (*P*<0.001, *P*<0.001). N-MID is positive correlation with ALP (*P*=0.04). There is no obvious correlation between other indicators.


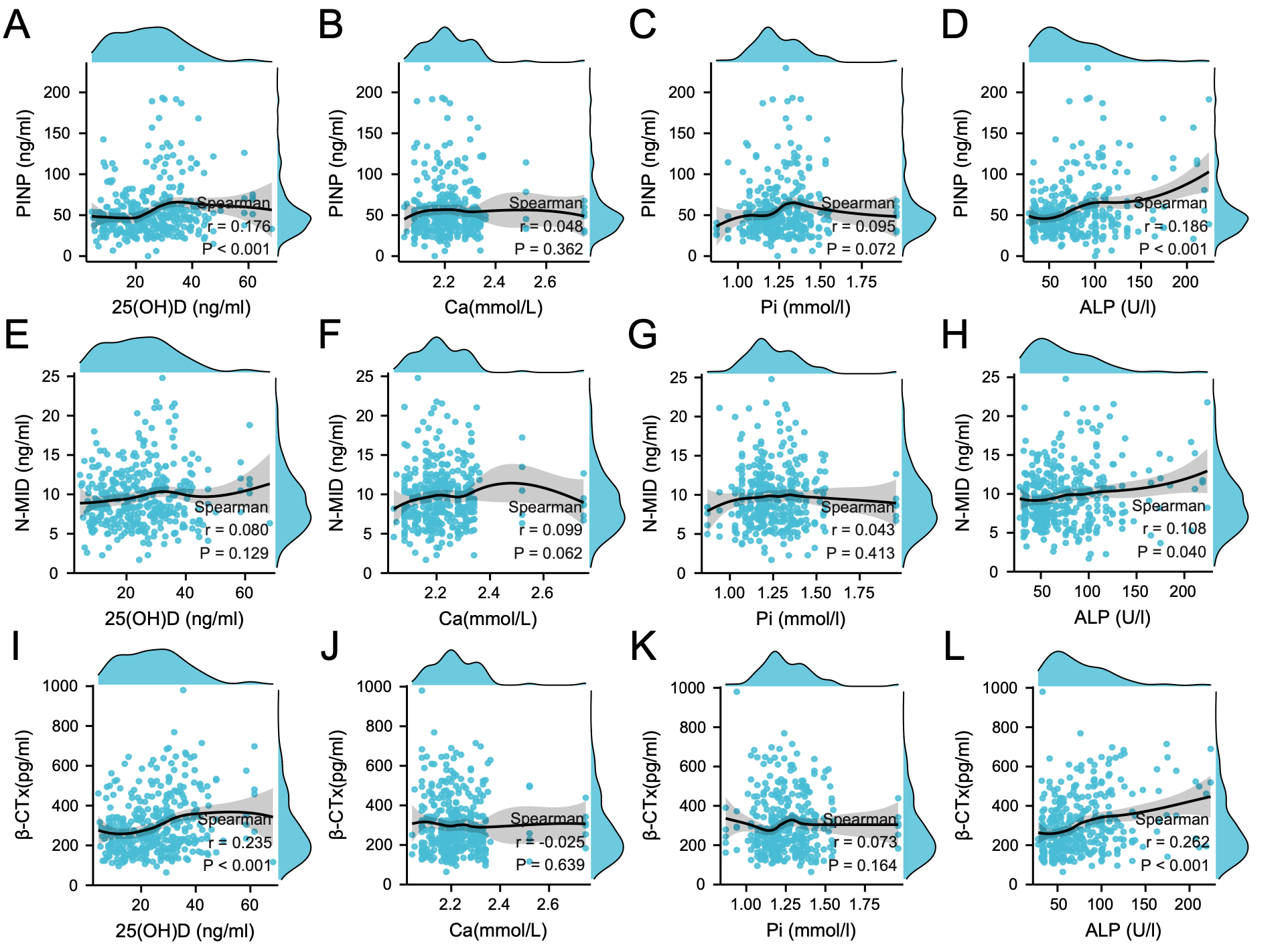


**Figure S1**. **Scatterplots showing the relation between serum concentrations of BTMs and biochemical indices in healthy pregnant women.** (A) PINP in relation to 25(OH)D. (B) PINP in relation to Ca. (C) PINP in relation to Pi. (D) PINP in relation to ALP. (E) N-MID in relation to 25(OH)D. (F) N-MID in relation to Ca. (G) N-MID in relation to Pi. (H) N-MID in relation to ALP. (I) β-CTX in relation to 25(OH)D. (J) β-CTX in relation to Ca. (K) β-CTX in relation to Pi. (L) β-CTX in relation to ALP.
